# Impact of Social Determinants on Melanoma Outcomes in Canada: A Single‐Centre Retrospective Study

**DOI:** 10.1002/jso.70069

**Published:** 2025-08-28

**Authors:** Aliyah King, Olivier Brandts‐Longtin, Chandy Somayaji, James Ted McDonald, Heidi Li, Katherine Aw, Rebecca Lau, Alex Lee, Boaz Wong, Elysia Grose, Ahmad Abed, James Stevenson, Rahat Sheikh, Stephanie Johnson‐Obaseki, Carolyn Nessim

**Affiliations:** ^1^ Faculty of Medicine University of Ottawa Ottawa Ontario Canada; ^2^ Ottawa Hospital Research Institute The Ottawa Hospital Ottawa Ontario Canada; ^3^ New Brunswick Institute for Research, Data, and Training University of New Brunswick Fredericton NB Canada; ^4^ Department of Political Science University of New Brunswick Fredericton NB Canada; ^5^ Division of Dermatology University of Ottawa Ottawa Ontario Canada; ^6^ Division of General Surgery, Department of Surgery University of Ottawa Ottawa Ontario Canada; ^7^ Department of Otolaryngology—Head and Neck Surgery University of Ottawa Ottawa Ontario Canada

**Keywords:** melanoma, social determinants, socioeconomic status, surgical oncology

## Abstract

**Introduction:**

Socioeconomic status (SES) and distance to nearest hospital are known social determinants impacting melanoma survival; however, few studies have investigated the effect in a universal healthcare setting, like Canada.

**Materials and Methods:**

This retrospective study at The Ottawa Hospital (1999–2023) investigated SES and distance lived from the hospital on overall survival, recurrence time, and stage at presentation in melanoma surgical patients. Income quintiles (InQs) were determined using postal codes linked to 2016 census data, and logistic regressions were conducted for the highest and lowest InQs.

**Results:**

Of 959 patients, 277 were in the highest InQ group (mean age: 64; 57% males) and 114 were in the lowest (mean age: 60; 48% males). Higher InQ was significantly associated with lower odds of stage II‐IV disease at presentation (*p* = 0.004, odds ratio: 0.865, 95% CI 0.784 to 0.954), but not with overall survival, recurrence time, or stage III‐IV disease. Distance had no significant impact on outcomes. Female sex was protective against recurrence time (*p* = 0.020, hazard ratio: 0.705), stage II‐IV (*p* = 0.049, odds ratio: 0.766, 95% CI: 0.587, 0.999), and III‐IV (*p* = 0.009, odds ratio: 0.670, 95% CI: 0.496, 0.904) disease.

**Conclusion:**

Higher SES reduced stage II‐IV risk without affecting survival, stage III‐IV risk, or recurrence time. Distance to nearest hospital had no significant effect. Females had longer time to recurrence and lower odds of advanced disease. Future research should explore potential educational and primary care barriers that may contribute to advanced stages in lower InQ populations.

**Abbreviations**
CCI: Charlson Comorbidity Index
InQ: Income quintile

## 1 Introduction

Despite melanoma constituting only 10% of all skin cancers, it is responsible for 80% of skin cancer‐related deaths [1]. Incidence and mortality rates of melanoma are rising, making it the 8th most common cancer in males and 7th most common in females in Canada [2, 3, 4]. Among patients aged 30 to 49 years old, melanoma is the 4th most common cancer in Canada [4]. Certain provinces, including Ontario, exhibit higher melanoma incidence rates than the national average [2].

While ultraviolet radiation remains the primary risk factor, the burden of melanoma has been described by others as being correlated with certain social determinants of health, including socioeconomic status (SES) [5, 6]. Higher‐income patients have also been shown to exhibit higher age‐standardized melanoma incidence rates [6]. Despite Canada's publicly funded healthcare system, our group's recent systematic review, spanning all Canadian provinces from 1979 to 2012, supported the notion that higher SES is associated with increased melanoma incidence, although with thinner tumours and a better prognosis [7]. Conversely, lower SES is correlated with later‐stage disease at presentation and greater mortality [7].

Distance to diagnosing provider has been shown to be a more complete measure of access to melanoma diagnosis compared to rurality, socioeconomic status, and provider supply [8]. Studies on the association between travel distance to healthcare providers and melanoma outcomes indicate that increased distance can delay screening, detection, and treatment, resulting in more advanced stage at diagnosis [8]. Increased distance to care has also been associated with increased Breslow depth and greater odds of metastatic disease at presentation in melanoma patients [9].

Given these findings, one might question the relevance of using income quintiles (InQ) as a measure of socioeconomic status in our study. However, income quintiles provide valuable insights into the economic context of patients, which may influence healthcare access and treatment decisions in ways that distance alone cannot capture. Therefore, this single‐centre retrospective study aimed to identify how SES, as defined by income quintile, affects melanoma outcomes in the Canadian healthcare system, specifically at The Ottawa Hospital. Overall survival, disease‐free survival, time to recurrence, and tumour stage at presentation were investigated. Secondary outcomes included distance lived from the hospital, age, sex, and comorbidity index.

## 2 Methods

This retrospective cross‐sectional study included all patients who underwent surgical treatment for melanoma at The Ottawa Hospital, a tertiary care center affiliated with the University of Ottawa, between 1999 and 2023. Inclusion criteria included biopsy‐proven melanoma, resulting in surgical treatment. Patients with other primary cancers were excluded to ensure no effect on survival. Local ethics board approval was granted.

The primary outcome of interest were area‐level income quintiles, and these were determined based on patient postal codes mapped to Statistics Canada Dissemination Areas (DA) to which income quintiles at the DA level were assigned from relevant pretax household income data drawn from the 2016 Census of Canada. Postal codes were also used to generate an estimate of travel distance to the hospital. The highest and lowest quintiles were pre‐specified before analysis. Other covariates collected included patient age, sex, Charlson Comorbidity Index (CCI), and tumour histological characteristics. Cancer outcome variables included melanoma stage at presentation, survival, and time to recurrence. Multivariate analyses were chosen based on clinical expertise. Melanoma stages were grouped either as stage I‐II versus stage III‐IV or as stage I versus stages II‐IV. These groupings account for melanoma's unique risk profile, where high‐risk stage II melanoma can have a prognosis comparable to or worse than stage III [10]. The secure web‐based REDCap database was used, which adhered to the regulations outlined in Canadian personal health governance laws [11].

Quantitative analyses were conducted by a senior data analyst. Patient postal codes were used to link cases to neighbourhood‐level average household income quintiles (InQs) before tax on a 1‐5 categorical scale: (1) lowest, (2) medium‐low, (3) middle, (4) medium‐high, and (5) highest quintile [12]. Significance of differences was based on analyses that compared patients in the lowest InQ to those in the highest InQ. Although descriptive comparisons focused on the lowest and highest income quintiles, all five income quintiles were included as categorical variables in multivariate models and in Kaplan‐Meier survival analyses for both overall and disease‐free survival. Distance, CCI, type of melanoma, palpable nodes at presentation, pathology documentation (i.e., lymphovascular invasion and ulceration), sentinel lymph node biopsy, metastasis, lymph node dissection, staging, adjuvant therapy, and mutational status were also compared across the lowest and highest InQs. Cox Proportional Hazard duration models with hazard ratios were used to determine time to survival and time to recurrence as a function of age, sex, CCI, InQs, and distance to the hospital. Logistic regression analyses, with odds ratio estimates, on stage at presentation as a function of age, sex, CCI, InQs, and distance to the hospital were performed.

Survival curves were created for overall survival and disease‐free survival by InQs with product‐limit survival estimates, including the number of patients at risk. The start date for disease‐free survival, overall survival, and time to recurrence was the date of first surgery. The end date for disease‐free survival was the first evidence of recurrence, date of death from any cause, or date of last follow‐up, whereas the end date for overall survival was the date of death from any cause or last follow‐up. The end date for time to recurrence was the date of the first evidence of recurrence. A threshold for statistical significance was set to *p* < 0.05. Given the number of comparisons conducted, we acknowledge the increased risk of Type I error. As this was an exploratory study, no formal corrections for multiple comparisons (e.g., Bonferroni adjustment) were applied. Consequently, p‐values, particularly those near the 0.05 threshold, should be interpreted with caution.

## 3 Results

### 3.1 Population Descriptives

Of the 959 patients undergoing melanoma surgery, there were 114 patients in the lowest InQ (mean age 60 [SD 17]; 52% females) and 277 in the highest InQ (mean age 64 [SD 14]; 57% males). There was no significant difference in age (*p* = 0.079), sex (*p* = 0.110), CCI (*p* = 0.090) between the two InQ groups. CCI scores were an average of 3 for both InQ groups. The mean distance from the hospital was 85 km (median 45 km; SD 300) for the lowest InQ and 29 km (median 16 km; SD 47) for the highest InQ (*p* = 0.050).

Increased age predicted worse survival outcomes (*p* < 0.0001, hazard ratio: 1.028), decreased time to recurrence (*p* = 0.023, hazard ratio: 1.013), and lower likelihood of stage I at presentation (*p* = 0.004, odds ratio: 0.984, 95% CI: 0.973, 0.995). Age was not significantly associated with stage II‐IV disease at presentation.

Female sex predicted an increased time to recurrence (*p* = 0.020, hazard ratio: 0.705), lower likelihood of stage III‐IV at presentation (*p* = 0.009, odds ratio: 0.670, 95% CI: 0.496, 0.904), and a lower likelihood of stage II‐IV at presentation (*p* = 0.049, odds ratio: 0.766, 95% CI: 0.587, 0.999). Sex was not significantly associated with overall survival.

Descriptives of melanoma type, palpable nodes presence, lymphovascular invasion presence, sentinel lymph node biopsy rates, lymph node dissection rates, staging at diagnosis, ulceration presence, and adjuvant therapy rates by income quintile are presented in Tables 1, 2, 3. A significantly higher proportion of patients in the lower InQ group had ulceration *(p* = 0.010). Additionally, there was a significant difference in the type of adjuvant therapy administered between the InQs (*p* = 0.020), with immunotherapy (9.920% difference) and targeted therapy (8.990% difference) more frequently used in the higher InQ population, and radiation therapy (18.050% difference) more common in the lower InQ population. The other variables did not show significant differences between the two groups.

**Table 1:** Melanoma type, palpable nodes, lymphovascular invasion, sentinel lymph node biopsy, and lymph node dissection by income quintile.

	Lowest InQ n (%)	Highest InQ n (%)
Type of Melanoma (*p* = 0.38)		
Acral lentiginous	2 (1.85%)	4 (1.47%)
Acral lentiginous, NOS	0 (0%)	1 (0.37%)
Amelanotic	1 (0.93%)	0 (0%)
Desmoplastic	1 (0.93%)	11 (4.03%)
Lentigo maligna melanoma	3 (2.78%)	15 (5.49%)
Mucosal	0 (0%)	3 (1.1%)
Nodular	34 (31.48%)	55 (20.15%)
Nodular, amelanotic	0 (0%)	1 (0.37%)
NOS	16 (14.81%)	31 (11.36%)
Not reported	5 (4.63%)	13 (4.76%)
Other	2 (1.85%)	8 (2.93%)
Spitzoid melanoma	1 (0.93%)	1 (0.37%)
Superficial spreading	43 (39.81%)	130 (47.62%)
Palpable nodes present *(p* = 0.23)	13 (11.5%)	21 (7.66%)
Lymphovascular invasion present (*p* = 0.5)	0 (0%)	4 (1.47%)
SLNB (*p* = 0.17)	91 (79.82%)	238 (85.92%)
SLNB containing metastasis (*p* = 0.16)	13 (14.29%)	40 (16.88%)
Lymph node dissection (*p* = 0.39)	24 (21.05%)	48 (17.33%)

Abbreviations: InQ, income quintile; NOS, not otherwise specified; SLNB, sentinel lymph node biopsy.

**Table 2:** T‐staging, ulceration, and overall melanoma stage at the time of diagnosis by income quintile.

	Lowest InQ n (%)	Highest InQ n (%)
T staging (*p* = 0.43)		
T0, no evidence of primary tumor, primary site of tumor is unknown	4 (3.54%)	5 (1.81%)
T1a, 0.8 mm, without ulceration	5 (4.42%)	19 (6.86%)
T1b, 0.8 mm, with ulceration; 0.8‐1.0 mm, with or without ulceration	21 (18.58%)	61 (22.02%)
T2a, > 1.0–2.0 mm, without ulceration	26 (23.01%)	80 (28.88%)
T2b, > 1.0–2.0 mm, with ulceration	3 (2.65%)	9 (3.25%)
T3a, > 2.0–4.0 mm, without ulceration	12 (10.62%)	32 (11.55%)
T3b, > 2.0–4.0 mm, with ulceration	9 (7.96%)	16 (5.78%)
T4a, > 4.0 mm, without ulceration	4 (3.54%)	12 (4.33%)
T4b, > 4.0 mm, with ulceration	25 (22.12%)	35 (12.64%)
Tis, melanoma in situ	2 (1.77%)	4 (1.44%)
Unable to assess A, B, C status	2 (1.77%)	4 (1.44%)
Ulceration present (*p* = 0.01)^a^	35 (32.11%)	56 (20.59%)
Overall melanoma stage (*p* = 0.28)		
Stage 0	2 (1.75%)	3 (1.08%)
Stage IA	22 (19.3%)	70 (25.27%)
Stage IB	25 (21.93%)	72 (25.99%)
Stage IIA	10 (8.77%)	31 (11.19%)
Stage IIB	9 (7.89%)	16 (5.78%)
Stage IIC	13 (11.4%)	13 (4.69%)
Stage IIIA	5 (4.39%)	11 (3.97%)
Stage IIIB	4 (3.51%)	16 (5.78%)
Stage IIIC	17 (14.91%)	28 (10.11%)
Stage IIID	4 (3.51%)	10 (3.61%)
Stage IV	0 (0%)	3 (1.08%)
Unable to stage	3 (2.63%)	4 (1.44%)

Abbreviation: InQ, income quintile.

a Significant.

**Table 3:** Adjuvant rates by income quintile.

	Lowest InQ n (%)	Highest InQ n (%)
Discussion about adjuvant therapy *(p* = 0.21)		
None offered, not needed	71 (62.28%)	184 (66.67%)
No effective therapy at the time	2 (1.75%)	5 (1.81%)
Eligible but not suitable	2 (1.75%)	10 (3.62%)
Offered but declined	7 (6.14%)	11 (3.99%)
Offered and accepted	32 (28.07%)	66 (23.91%)
Decision about adjuvant therapy (*p* = 0.47)		
No adjuvant offered therapy offered to patient (does not require adjuvant therapy)	71 (62.28%)	184 (66.67%)
No effective adjuvant therapy at the time	2 (1.75%)	5 (1.81%)
Patient was eligible for adjuvant therapy but deemed not suitable for adjuvant therapy	2 (1.75%)	10 (3.62%)
Patient was offered adjuvant and declined adjuvant therapy	7 (6.14%)	11 (3.99%)
Patient was offered adjuvant therapy and accepted	32 (28.07%)	66 (23.91%)
Type of adjuvant therapy (*p* = 0.02)^a^		
Chemotherapy	0 (0%)	1 (1.37%)
Chemotherapy, immunotherapy	0 (0%)	1 (1.37%)
Chemotherapy, immunotherapy, radiation	0 (0%)	2 (2.74%)
Immunotherapy	12 (35.29%)	33 (45.21%)
Immunotherapy, radiation	5 (14.71%)	12 (16.44%)
Radiation	8 (23.53%)	4 (5.48%)
Targeted	3 (8.82%)	13 (17.81%)
Targeted, immunotherapy	0 (0%)	1 (1.37%)
Targeted, immunotherapy, radiation	3 (8.82%)	0 (0%)
Targeted, radiation	1 (2.94%)	0 (0%)
No adjuvant therapy	2 (5.88%)	6 (8.22%)

Abbreviation: InQ, income quintile.

a Significant.

### 3.2 Duration Models and Logistical Regressions

Over the period of study, the majority of patients survived (794 [83%]). Of 953 patients, 739 (78%) did not recur. InQ and distance from the hospital were not significantly associated with overall survival, stage III‐IV disease at presentation, nor time to recurrence. Higher InQ was significantly associated with lower odds of being diagnosed at a higher melanoma stage (stages II‐IV) compared to stage I (*p* = 0.004, odds ratio: 0.865, 95% CI 0.784 to 0.954). Distance from the hospital was not significantly associated with stage II‐IV disease at presentation.

**Figure 1:**
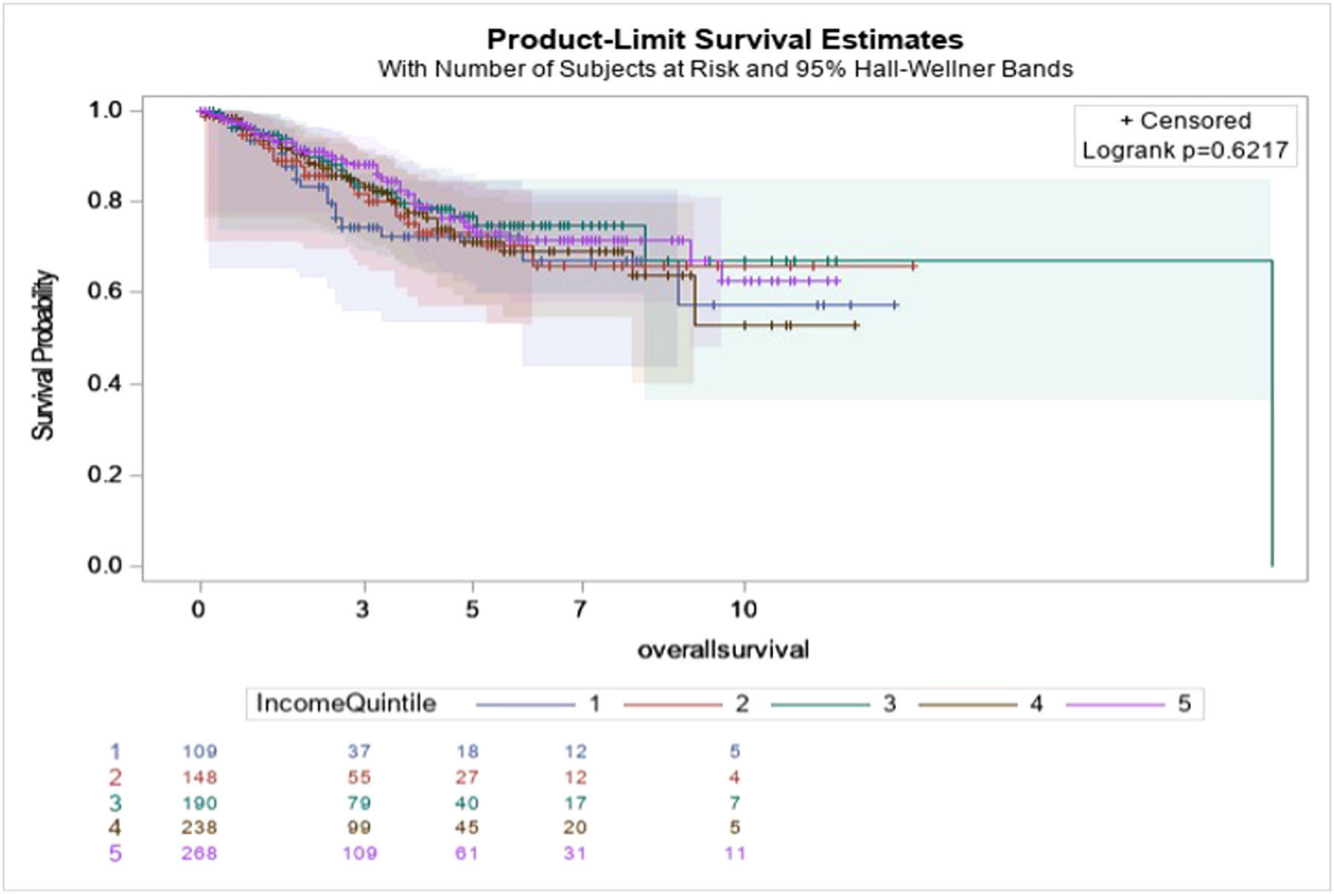
Overall survival in years by income quintile.

**Figure 2:**
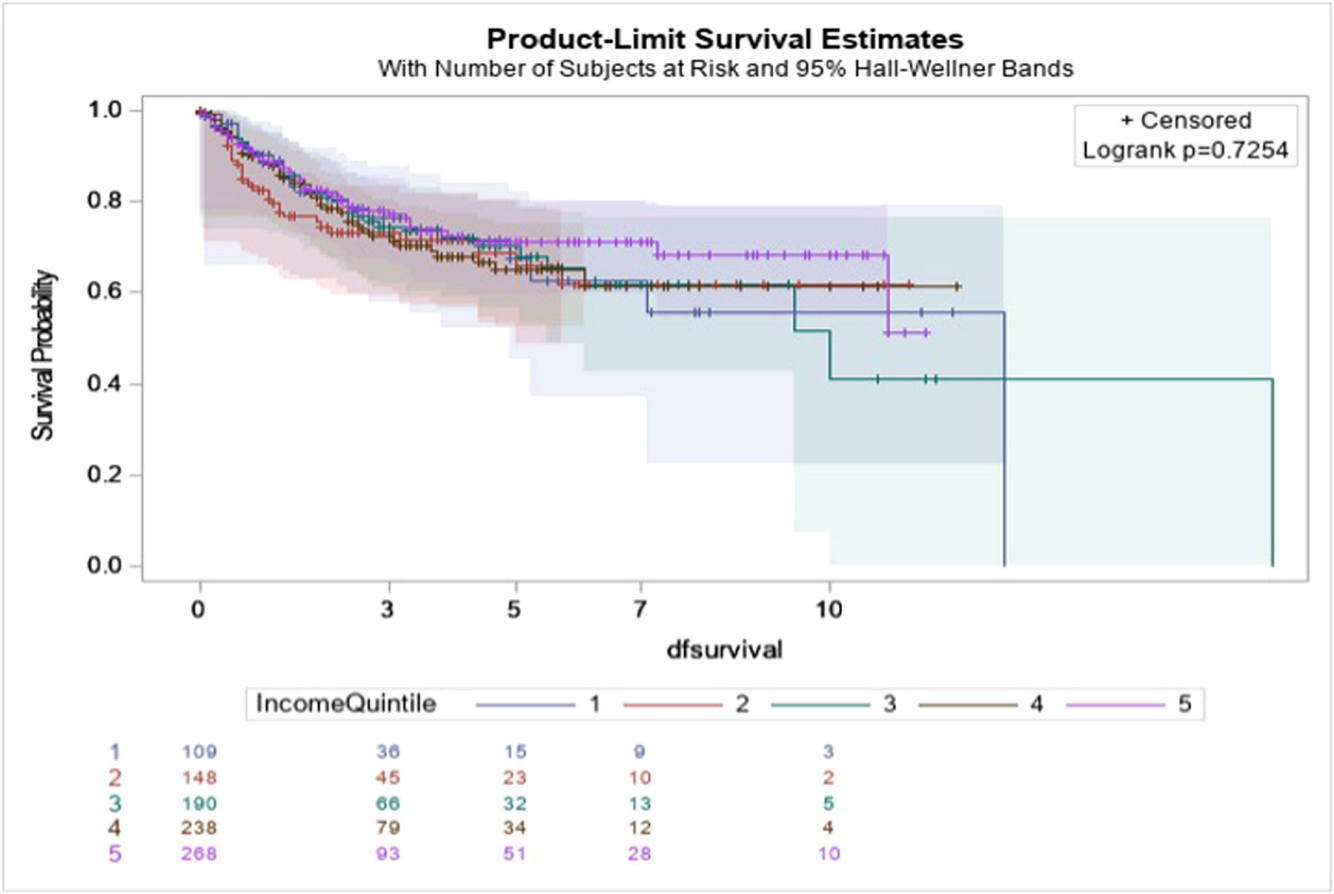
Disease‐free survival in years by income quintile.

Overall and disease‐free survival by InQs are displayed in Figures 1 and 2.

## 4 Discussion

Discovering and understanding the effects of SES and distance from treatment centers on melanoma outcomes is of great importance to melanoma management and delivering equitable care. In our Eastern Ontario cohort, we found that InQs were not associated with overall survival, time to recurrence, or stage III‐IV disease, and distance from the hospital had no significant impact on survival, stage III‐IV disease at presentation, nor time to recurrence. While InQs did not significantly affect the number of patients on adjuvant therapy (*p* = 0.470), they did influence the type of adjuvant therapy administered (*p* = 0.020). The implication, then, is in our cohort lower InQ patients appear more likely to present at stage II‐IV disease (*p* = 0.004) with ulcerated tumours (*p* = 0.010); however, the influence on survival outcomes and recurrence may be mediated by other factors. This may be due to the fact that in Canada and in Eastern Ontario, specialized care is centralized to the academic centers and the community hospitals appropriately refer patients with melanoma to The Ottawa Hospital Cancer Center thus providing equal opportunity for care. Despite higher stage and more aggressive disease features in lower InQ and further away populations, these patients seem to get an equal access to surgery and adjuvant therapies to have equivalent survival.

Our study highlighted the already well‐supported finding that higher CCI, increased age, and male sex are associated with worse survival and decreased time to recurrence in surgical melanoma patients. Older patients are more likely to develop locoregional recurrences and likely have worse overall health and ability to fight diseases, leading to worse melanoma outcomes [13, 14]. Although younger patients are more likely to have lymph node metastases, the prognosis is overall better [13]. Female sex was also a significant predictor of increased time to recurrence and decreased stage at presentation, which has been supported by findings from multiple publications and may be partly due to differences in health‐seeking behavior, though the underlying mechanisms are likely multifactorial [15, 16].

The analysis of distance from the hospital revealed no significant impact on melanoma overall survival, time to recurrence, or stage at presentation. Despite the 56 km difference between the InQ groups, there was a similar distribution of melanoma types (*p* = 0.380), staging (*p* = 0.280), palpable nodes (*p* = 0.230), lymphovascular invasion (*p* = 0.500), sentinel node biopsies (*p* = 0.170), nodal dissection (*p* = 0.390), mutation status testing (*p* = 0.680), and adjuvant therapy discussions (*p* = 0.210) and decisions (*p* = 0.470). This suggests that in Eastern Ontario, with a publicly funded healthcare system, care is well regionalized across distances. In our opinion, we believe that InQ may potentially be playing a more pronounced role in education of when to consult for an opinion about an abnormal skin lesion thus presenting with more advanced disease at a later stage or potentially less access to dermatology in distant areas.

Desmoplastic melanoma, a subtype commonly associated with chronic sun exposure, was frequent among higher InQ patients. Data on tumour anatomic location and patient birthplace or immigration status were not captured in our database, but represent important factors for future studies investigating environmental or occupational exposure differences across SES groups. There has been data to suggest that earlier stage melanoma occurs more often in higher InQ patient potentially because they have the income to travel south or own a dwelling in the south leading to more sun exposure.

Although all patients were treated within a centralized multidisciplinary cancer center, differences emerged in the type of adjuvant therapy administered. Higher InQ patients more often received immunotherapy or targeted therapy, while radiation therapy was more common in lower InQ patients. These differences may reflect variation in mutation status, comorbidities, treatment eligibility, or patient preferences potentially not wanting to travel from far to receive systemic therapy, though these were not uniformly captured in our data. It is also important to consider that the study period (1999–2023) spans major shifts in systemic therapy, including the transition from interferon‐based immunotherapy to modern checkpoint inhibitors. Our data set did not stratify outcomes by therapeutic era, and some of these variations may reflect temporal differences in treatment availability.

Our cohort includes only patients who underwent surgery, excluding those with unresectable stage III/IV melanoma at presentation. This may underestimate the true incidence of late‐stage disease and limits generalizability to the entire melanoma population. Additionally, melanoma in situ was underrepresented, likely because many cases are managed in dermatologic or community settings. Furthermore, although we focused on the highest and lowest quintiles to highlight disparities, this limits interpretation of SES as a continuous variable. InQ by postal code may have also missed some InQ details that could be found at the individual rather than neighbourhood level. Lastly, as a single‐centre retrospective design, additional limitations include small cohort size, missing data, patient misclassification, and how reporting of cancer information is not legally mandated and thus conducted passively. Future multicenter studies with granular individual‐level data will be important to validate and expand upon these findings.

Overall, we saw that lower InQ patients appear to be presenting with more advanced‐stage and more ulceration, which could be the result of a patient's awareness to consult a doctor for a lesion or greater difficulty in accessing a primary care practitioner or dermatologist. Further research should focus on identifying potential educational and primary care challenges that could contribute to the increased likelihood of advanced‐stage melanoma in lower InQ populations. As early intervention is imperative for survival, improving access to primary care practitioners and dermatologists for early diagnosis, coupled with understanding patients’ willingness to seek help, could lead to improved outcomes and more equitable care for all patients [17].

## 5 Conclusion

Higher InQ is a significant predictor of decreased stage at presentation. However, neither InQ, or distance from treatment centre, appear to affect overall survival or time of recurrence. Lower InQ populations appear to have an increased likelihood of ulceration at presentation. Further research should aim to identify potential educational and primary care barriers that may contribute to the higher likelihood of advanced‐stage melanoma in lower InQ populations.

## Consent

The authors have nothing to report.

## Conflicts of Interest

The authors declare no conflict of interest.

## Synopsis

This retrospective study examines the impact of socioeconomic status (SES) and hospital proximity on melanoma outcomes in Canada's universal healthcare system, analyzing patients treated at The Ottawa Hospital from 1999 to 2023. Higher SES was associated with a lower likelihood of stage II‐IV melanoma at presentation, while distance to the nearest hospital had no effect on outcomes. Our findings highlight the role of SES in melanoma diagnosis and may help guide interventions to improve early detection and care for lower SES groups.

## Data Availability

The data that support the findings of this study are available from the corresponding author upon reasonable request.
